# OrgaQuant: Human Intestinal Organoid Localization and Quantification Using Deep Convolutional Neural Networks

**DOI:** 10.1038/s41598-019-48874-y

**Published:** 2019-08-28

**Authors:** Timothy Kassis, Victor Hernandez-Gordillo, Ronit Langer, Linda G. Griffith

**Affiliations:** 1 Department of Biological Engineering, Massachusetts Institute of Technology, Cambridge, MA USA; 2 Department Electrical Engineering and Computer Science, Massachusetts Institute of Technology, Cambridge, MA USA

**Keywords:** Optical imaging, Software, Cell growth, Organogenesis, Intestinal stem cells

## Abstract

Organoid cultures are proving to be powerful *in vitro* models that closely mimic the cellular constituents of their native tissue. Organoids are typically expanded and cultured in a 3D environment using either naturally derived or synthetic extracellular matrices. Assessing the morphology and growth characteristics of these cultures has been difficult due to the many imaging artifacts that accompany the corresponding images. Unlike single cell cultures, there are no reliable automated segmentation techniques that allow for the localization and quantification of organoids in their 3D culture environment. Here we describe OrgaQuant, a deep convolutional neural network implementation that can locate and quantify the size distribution of human intestinal organoids in brightfield images. OrgaQuant is an end-to-end trained neural network that requires no parameter tweaking; thus, it can be fully automated to analyze thousands of images with no user intervention. To develop OrgaQuant, we created a unique dataset of manually annotated human intestinal organoid images with bounding boxes and trained an object detection pipeline using TensorFlow. We have made the dataset, trained model and inference scripts publicly available along with detailed usage instructions.

## Introduction

Many of today’s biological discoveries have been made using *in vitro* cell culture systems. These systems allow researchers to conduct hypothesis-driven research on a specific cell type to gain a mechanistic understanding of its various processes as well as for testing drugs in pharmaceutical research. Conventional *in vitro* cultures have either used primary cells or immortalized cell lines plated on 2D surfaces. While these offer utility, they are not very faithful in recapitulating the complex physiological environment^1^ and are rarely predictive of *in vivo* behavior. Recently there has been a rise in what is called ‘organoid’ cultures^2–5^. Organoids are multicellular spheroids that are derived from either a primary donor or stem cells. In many regards, they resemble their parent organ in both functionality and cellular composition. For example, several well-received studies have demonstrated the establishment of organoids from the gut^6–8^, pancreas^9–11^, brain^12^, liver^13^, and endometrium^14^, among others. Organoids are fast becoming the ideal model system for understanding development, investigating physiology, and for drug testing^3,15,16^.

**Figure 1:**
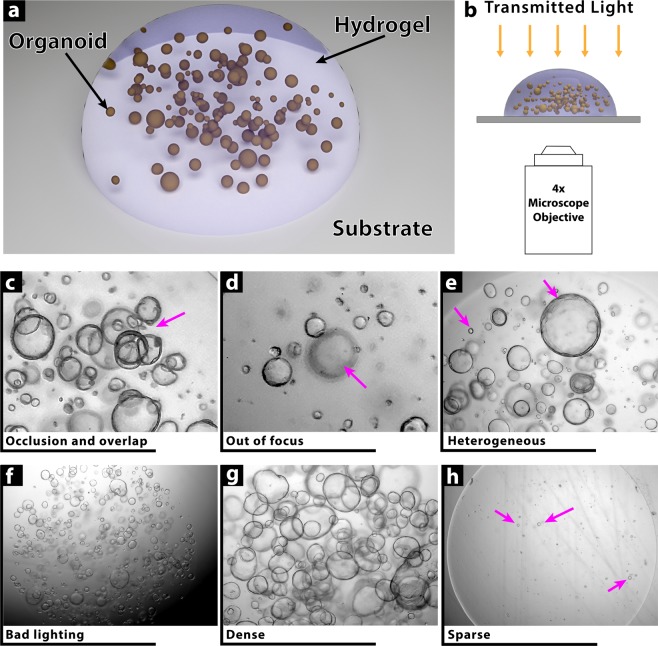
Challenges in Imaging Organoid Cultures. (**a**) Organoids are typically cultured in a 3D hydrogel droplet formed from either naturally-derived or synthetic extracellular matrices. The droplet sits on a transparent substrate such as the polystyrene bottom of a multi-well culture plate. Each droplet can have anywhere from zero to several hundred organoids. (**b**) Organoid droplets are imaged using low magnification objectives to efficiently capture a wide field of view using bright-field modalities. (**c**–**h**) As a result of the culture and imaging methods, there are a variety of imaging artifacts that render conventional segmentation and image processing techniques unreliable. These artifacts include occlusion and overlap (**c**), out of focus organoids (**d**), heterogeneous size distribution (**e**), sub-optimal lighting conditions (**f**), very dense (**g**) or very sparse (**h**) cultures.

Obtaining successful organoid cultures that recapitulate the *in vivo* functionality and cellular composition of the target organ requires a tremendous amount of optimization by researchers. Deriving these organoids requires embedding them in biological hydrogels that provide the necessary extracellular microenvironment including growth factors and structural support, and monitoring them over time (days to weeks). Quantifying morphological changes, such as size and shape, as a function of growth or stimulation conditions, is fundamental for their use in research. Currently, the standard differentiation and culture protocol is to form these organoids inside a gel droplet (Fig. 1a) that sits on a substrate which is typically the polystyrene bottom of a cell culture multi-well plate or petri dish. To monitor these cultures, the droplet is imaged using a low magnification objective in brightfield (Fig. 1b).

The obtained images suffer from numerous imaging artifacts that make conventional image processing techniques extremely difficult. The artifacts include organoid occlusion and overlap, out of focus spheroids, large heterogeneity in size and shape, adverse lighting conditions, and highly dense or highly sparse organoid distributions (Fig. 1c–h). Manually measuring and counting these organoids is a very inefficient process as typically there are hundreds of images that need to be quantified with tens to hundreds of organoids per image. As a result, most studies either score by hand a limited number of images or use the images as representative samples and are not quantified.

Recently *Borten et al*. released an elegant open-source software package, OrganoSeg^17^, that addresses some of these challenges, but still relies on conventional image processing techniques and requires tweaking of multiple parameters for any given set of images with similar optical conditions. Instance-based detection using deep convolutional neural networks, however, offers an auspicious approach to address this and similar problems. Building on Tensorflow^18^, Google has recently released an object detection API^19^ that makes configuring, training, testing, and running various object detection neural architectures substantially more accessible to scientists than before. Utilizing the object detection API, here we present a practical open-source implementation, OrgaQuant, which allows any user to automatically detect and localize a human intestinal organoid within a typical bright-field image. Based on the idea of transfer learning^20^, we take a pre-trained neural network and further train it on organoid images to achieve very high precision results in drawing a bounding box around each organoid. Once a bounding box is determined, downstream processing allows further quantification, including size and shape measurements. Using the algorithm does not require any parameter tuning and runs autonomously on all images in a given folder and sub-folders while being robust against the various imaging artifacts described in Fig. 1c–h. We have made the training dataset, trained model, and inference scripts publicly available along with detailed usage instructions. Additionally, a ready-to-run cloud implementation is available on www.scipix.io.

## Results

### A new bounding-box annotated image dataset of bright-field human intestinal organoids

**Figure 2:**
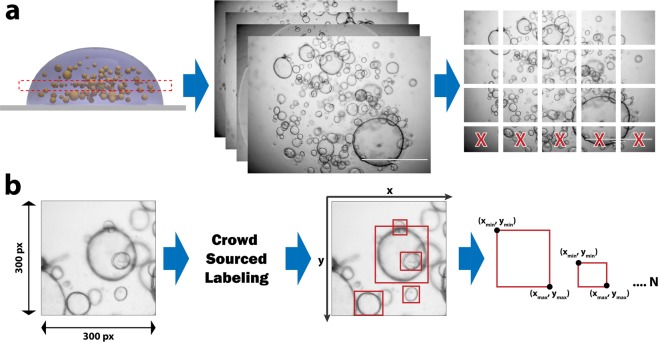
Dataset Creation Workflow. (**a**) A single image is captured using a 4x objective which is then divided into 300 × 300 (and 450 × 450) pixel patches to make annotations less overwhelming to our crowdsourcing community and to fit within GPU memory of our neural network training computational hardware. (**b**) The patches were then distributed on a crowdsourcing platform called CrowdFlower, now known as Figure Eight, along with detailed instructions on what to annotate. Several redundancy techniques were used to assure quality, as described in the methods. The resulting dataset includes box coordinates (x_min_, y_min_, x_max_, y_max_) for each organoid in an image.

Since there are no publicly available datasets for our model training, we created a new unique dataset comprising a total of approximately 14,240 organoids each annotated with bounding box coordinates (Fig. 2). Please see the methods section for dataset-creation workflow. The full dataset, including the images and annotations, are publicly available at https://osf.io/etz8r under an MIT license.

### A fast, accurate, parameterless and fully automated algorithm for human intestinal organoid localization and quantification

**Figure 3:**
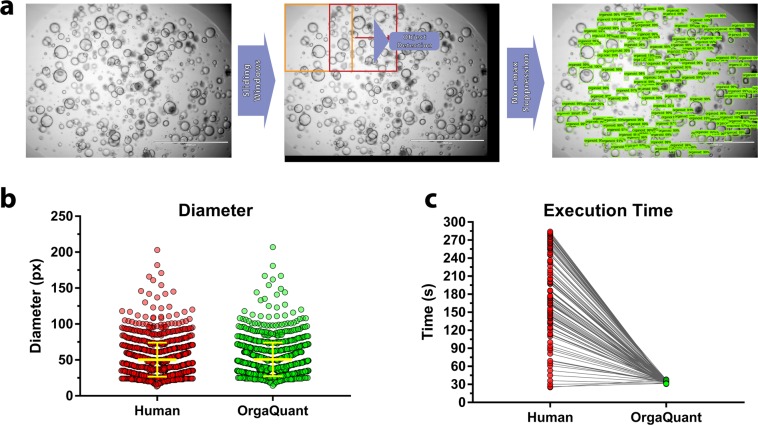
Automated Quantification of Organoids using OrgaQuant. (**a**) The inference script runs on individual patches (the size of which can be controlled by the user). To cover an entire image, a sliding window with overlap is used. The original input image is padded at the bottom and right edges (shown in black) in order to have an integer number of windows cover each of the horizontal and vertical sliding axes. All the results are automatically aggregated to provide a fully labeled image with the corresponding bounding box coordinates that can be used downstream for any type of processing that might be desirable by the researcher. (**b**) There is no difference between human and OrgaQuant measurements, N = 1,135 organoids. P = 0.35 (Welch’s unpaired t-test) (**c**) OrgaQuant is substantially faster compared to our trained human annotators and always has the same inference time per patch (around 30 seconds depending on the GPU used). N = 112 different humans.

OrgaQuant provides a quantification Jupyter Notebook file that can be run to quantify all images within a folder and sub-folders. The resulting output is a CSV file for each image containing the bounding box coordinates for each organoid, projected 2D area measurements as well as lengths of the major and minor axis of an organoid (which is assumed to be an ellipse). The inference script quantifies an input image by using a sliding window for which both the size and overlap can be set by the user if needed (Fig. 3a). The sliding window is used to circumvent GPU memory limitations if the entire high-resolution image was given as input. Organoids at the edge of each sliding window patch are ignored thus, an overlap between windows should be used. The output is a single image with all the aggregated labels. Both the labeled image and the CSV labels file are saved in the same folder as the original input image. OrgaQuant labeling quality is indistinguishable from that of humans (p = 0.35) for a given image set (Fig. 3b) with a mean average precision (mAP) of 80%, but is substantially faster and more consistent requiring only 30 sec/patch (on an NVIDIA Quadro P5000 GPU) vs. anywhere from 25 to 284 seconds for humans (Fig. 3c).

## Discussion

Object detection and localization is a complex problem in computer vision applications. It is especially tricky when fast detection performance is required. There have been several detection algorithms implemented to provide a balance between speed and accuracy. Two prevalent approaches are Single Shot Multibox Detector (SSD)^21^ and You Only Look Once (YOLO)^22^. While these are ideal for real-time detection, they accomplish speed by sacrificing accuracy. For OrgaQuant, we decided to implement Region Convolutional Neural Network (R-CNN) and specifically what is referred to as Faster R-CNN. Faster R-CNN can use a detection model based on several different architectures including ResNet 101^23^ and Inception v2^24^. Here we chose an architecture based on both Inception v2 and ResNet called Inception-ResNet-v2^25^ for which an implementation is provided with the TensorFlow object detection API. The model has been pre-trained on a box annotated COCO dataset^26^ and for our purpose, we fine-tuned the model by training it on our organoid dataset. Since Inception-ResNet models have many parameters it is important to use a very large dataset. To achieve this, we augmented the dataset as described in the methods section.

The resulting implementation of OrgaQuant can automatically localize an organoid within a brightfield image and label it with a bounding box. The cropped organoid image can, in turn, be used in any number of downstream image processing and analysis pipelines. Given the nature of our training set, the current model provided with this manuscript is only capable of accurately localizing spherical organoids (i.e. organoids with non-crypt-like structures). Here, we demonstrated the ability to measure the human intestinal organoid size, but an important byproduct of convolutional neural networks is that they extract features that can be used with various other machine learning algorithms. These features can be used, for example, to cluster similar organoid based on visual similarly or even detect subtle changes in organoid morphology in response to stimuli that cannot necessarily be detected with normal human vision.

Given the fact that each organoid is localized in 2D space, we can also track the individual growth kinetics of each organoid in a droplet over time. While we don’t explicitly use OrgaQuant for this, it is as easy as loading a time-lapse set of images in a folder and analyzing it. We believe OrgaQuant is a basis for many exciting and intelligent organoid quantification techniques and we look forward to working the organoid community to develop this open-source implementation further.

## Methods

### Intestinal organoid culture

De-identified tissue biopsies were collected from unaffected duodenum areas of children and adult patients undergoing endoscopy for gastrointestinal complaints. All experimental methods and protocols were approved by and carried out in accordance with the Institutional Review Board of Boston Children’s Hospital (IRB-P00000529). Informed consent was obtained from adult patients and from the legal guardians of the minor donors with assent from the patients which all were obtained at Boston Children’s Hospital. Tissue was digested in 2 mg/ml of collagenase I for 40 min at 37 °C followed by mechanical dissociation. Isolated crypts were resuspended in growth factor-reduced (GFR) Matrigel (Becton Dickinson) and polymerized at 37 °C. Organoids were grown in organoid expansion medium (OEM) consisting of Advanced DMEM/F12 supplemented with L-WRN conditioned medium (50% vol/vol, ATCC, cat. no. CRL-3276)^8^, glutamax, HEPES, murine epidermal growth factor (EGF, 50 ng/ml), N2 supplement (1×), B27 supplement (1×), human [Leu15]-gastrin I (10 nM), N-acetyl cysteine (1 mM), nicotinamide (10 mM), SB202190 (10 μM), A83-01 (500 nM), and Y-27632 (10 µM) as described^27,28^. Media was changed every two days and organoids were passaged every 4 days by incubating in Cell Recovery Solution for 40 min at 4 °C, followed by trypsin digestion for 5 min at 37 °C to obtain single cells. Single cells were seeded at a density of 25,000 cells in 25 µL of GFR Matrigel. For experiments involving the synthetic hydrogels, single cells were seeded at a density of 500 cells/µL. Three µL of cells suspension (Matrigel or synthetic hydrogels) were loaded in a 96-well plate an allowed to polymerase for 15–20 min at 37 °C. 100 µL of OEM was loaded in each well. Media was changed every two days.

### Image acquisition

Images of organoids suspended in gel droplets were acquired using a Thermo EVOS FL microscope with a 4x objective at days 4 and 6 of culture in normal bright-field mode. Images were saved as 8-bit TIFFs along with a scale bar. A single image was taken for a droplet. Since the organoids are suspended in the gel, the focus level was chosen to have the most organoids in focus as determined subjectively by the user. The resulting images were 1500 × 1125 pixels and were approximately 4.5 MB in size.

### Training dataset creation

There are no publicly available datasets for labeled organoid images. Instead, we created our own (Fig. 2). Each image (which was around 1500 × 1125 pixels) was divided into 300 × 300 pixel and 450 × 450 pixel patches. It was important to use patches because the original image was (1) too big to fit into GPU memory and (2) too difficult to label as it had hundreds of organoids. The patches were then labeled using a crowdsourcing platform (Crowdflower.com, now known as Figure-Eight) where the workers drew a bounding box around each organoid that was considered to be in focus (i.e., not having very blurry edges). The definition of what ‘in focus’ is very subjective, and there was no way to easily standardize that during the process of manual labeling. Each image was labeled by two different workers, and if there was less than 80% agreement (as defined by calculations of Intersection over Union (IoU) carried out by CrowdFlower), the image was presented to a third worker for futher annotation. The bounding boxes that were chosen for each image where an aggregate where a box is only chosen if there was 70% agreement between all workers. Detailed instructions and examples were provided to the workers who could only complete the task after a quality test they underwent. Additionally, each individual labeling task had a discrete test image to assure data integrity. The resulting dataset was composed of 1,750 image patches and a total of 14,242 aggregated bounding boxes. The dataset was randomly divided into training and test sets. Training had 13,004 boxes and test had 1,135. There were a total of 1,745 unique images that had at least one bounding box. The bounding box data was stored in a ‘.csv’ file where each row contained:

1. **filename**: the image name in which the bounding box is located
2. **width**, **height**: of the image patch (in our case we had two different patch sizes 300 × 300 and 450 × 450)
3. **class**: the label for the bounding box. ‘organoid’ was the only label we used.
4. **xmin, ymin, xmax, ymax**: define the coordinates of the bounding box where the origin (0,0) is located in the top left corner of the image.

### Hyperparameter selection and neural network training

While implementing a Faster R-CNN from scratch is no trivial task. The TensorFlow object detection API made is incredibly easy. While we will not reiterate the steps we took which are well documented on the TensorFlow API’s GitHub page. We will briefly describe the entire implementation and refer the user to our code for more details.

1. The dataset was created by breaking apart large microscope images of organoids into 300 × 300 and 450 × 450 pixel patches.
2. The patches were then uploaded to a Google Storage Bucket to make them accessible to our crowdsourced annotators.
3. An detailed instruction manual was written for the crowdsourcing platform called CrowdFlower.com, and a new job on the platform was set up to annotate the images using bounding boxes as defined by specific instructions.
4. The resulting ‘.csv’ file included the x_min_, y_min_, width and height of each bounding box. A small python script was written to change that to x_min_, y_min_, x_max_, and y_max_ as this is the preferred format for the helper scripts used below.
5. The ‘.csv’ file was broken into a training set and a test set.
6. A helper script provided by the API was then used to transform the data from .csv format into TFrecords (which is a TensorFlow data format used by the API).
7. A configuration script was then created where we specified the number of classes (in this case only one), augmentation strategy, data location…etc. We also had the option of specifying parameters relating to the Faster R-CNN architecture, but we decided to stick with the defaults as that seemed to work well during initial tests. The hyperparameters we adjusted were:

- The batch size used was one as anything larger did not fit into a single GPU memory.
- Total training steps of 200k with no stopping criteria
- We used an SGD optimizer with 0.9 momentum, and learning rate was adjusted to decrease with the number of steps as follows:

i. LR = 0.001 from step 0–50k
ii. LR = 0.0001 from step 50–80k
iii. LR = 0.00001 above 80k

8. The training was carried out on a cloud-based Windows Server 2016 instance on Paperspace.com and took around three days on a Quadro P5000 GPU with 16 GB of GPU RAM. The service used was Paperspace.com as it was cheaper than both AWS and Google Cloud (for GPU instances) at the time we trained.
9. TensorFlow comes with TensorBoard, which allowed us to observe the training loss as it was training and to calculate the mean average precision for the implementation (mAP) using the code-base provided by the API.

The main metric we used to evaluate the algorithm’s accuracy was the mean average precision (mAP). This metric is the gold standard for assessing object detection algorithms. The mAP was determined using a 10% held out test set that the training algorithm had not seen. To describe the metric in a bit more detail: The average precision refers to what fraction of the ground truth (manually annotated) bounding boxes were found by the algorithm. For example, if an image has two organoids (hence two bounding boxes) and the algorithm detects only one of them, then the average precision is 0.5 or 50%. If it detects both of them, then it would be 100%. The mAP is then the mean of all the precisions calculated across all the test images. Hence the closer the mAP to 100% the better is the algorithm. Note that in order to compare the bounding box created by the algorithm with the ground truth, it was assumed if there was 70% overlap (i.e., 0.7 intersection over union) then it was considered the same bounding box. While in some instances, it might be useful to have a metric that measures computational efficiency, here it was not a large concern as the implementation did not have to be fast. For example, no real-time detection was desired.

## Data Availability

All code for inference is available at https://github.com/TKassis/OrgaQuant, the trained model and full training dataset can be downloaded from https://osf.io/etz8r.

## References

[1] Jackson EL, Lu H (2016). Three-dimensional models for studying development and disease: moving on from organisms to organs-on-a-chip and organoids. Integr. Biol..

[2] Bredenoord AL, Clevers H, Knoblich JA (2017). Human tissues in a dish: The research and ethical implications of organoid technology. Science (80−.)..

[3] Schweiger PJ, Jensen KB (2016). Modeling human disease using organotypic cultures. Curr. Opin. Cell Biol..

[4] Clevers H (2016). Modeling Development and Disease with Organoids. Cell.

[5] Dutta D, Clevers H (2017). Organoid culture systems to study host–pathogen interactions. Curr. Opin. Immunol..

[6] Cruz-Acuña R (2017). Synthetic hydrogels for human intestinal organoid generation and colonic wound repair. Nat. Cell Biol..

[7] Múnera JO (2017). Differentiation of Human Pluripotent Stem Cells into Colonic Organoids via Transient Activation of BMP Signaling. Cell Stem Cell.

[8] Sato T (2009). Single Lgr5 stem cells build crypt-villus structures *in vitro* without a mesenchymal niche. Nature.

[9] Broutier L (2016). Culture and establishment of self-renewing human and mouse adult liver and pancreas 3D organoids and their genetic manipulation. Nat. Protoc..

[10] Grapin-Botton A (2016). Three-dimensional pancreas organogenesis models. Diabetes, Obes. Metab..

[11] Kim Y (2016). Islet-like organoids derived from human pluripotent stem cells efficiently function in the glucose responsiveness *in vitro* and *in vivo*. Sci. Rep..

[12] Serruya MD (2017). Connecting the brain to itself through an emulation. Front. Neurosci..

[13] Skardal A, Devarasetty M, Rodman C, Atala A, Soker S (2015). Liver-Tumor Hybrid Organoids for Modeling Tumor Growth and Drug Response *In Vitro*. Ann. Biomed. Eng..

[14] Turco MY (2017). Long-term, hormone-responsive organoid cultures of human endometrium in a chemically defined medium. Nat. Cell Biol..

[15] Shamir, E. R. & Ewald, A. J. Three-dimensional organotypic culture: experimental models of mammalian biology and disease. *Nat. Publ. Gr*. **15**, (2014). 10.1038/nrm3873 PMC4352326 25237826

[16] Skardal A, Shupe T, Atala A (2016). Organoid-on-a-chip and body-on-a-chip systems for drug screening and disease modeling. Drug Discov. Today.

[17] Borten MA, Bajikar SS, Sasaki N, Clevers H, Janes KA (2018). Automated brightfield morphometry of 3D organoid populations by OrganoSeg. Sci. Rep..

[18] Schapiro Anna C, Rogers Timothy T, Cordova Natalia I, Turk-Browne Nicholas B, Botvinick Matthew M (2013). Neural representations of events arise from temporal community structure. Nature Neuroscience.

[19] Huang, J. *et al*. Speed/accuracy trade-offs for modern convolutional object detectors. (2016).

[20] Esteva A (2017). Dermatologist-level classification of skin cancer with deep neural networks. Nature.

[21] Liu W (2016). SSD: Single shot multibox detector. In Lecture Notes in Computer Science (including subseries Lecture Notes in Artificial Intelligence and Lecture Notes in Bioinformatics).

[22] Redmon, J., Divvala, S., Girshick, R. & Farhadi, A. You Only Look Once: Unified, Real-Time Object Detection. *CVPR*, 10.1109/CVPR.2016.91 (2015).

[23] He, K., Zhang, X., Ren, S. & Sun, J. Deep Residual Learning for Image Recognition. 2016 IEEE Conf. *Comput. Vis. Pattern Recognit*. 770–778, 10.1109/CVPR.2016.90 (2016).

[24] Ioffe S, Szegedy C (2015). Batch Normalization: Accelerating Deep Network Training by Reducing Internal Covariate Shift. IEEE Trans. Very Large Scale Integr. Syst..

[25] Längkvist Martin, Karlsson Lars, Loutfi Amy (2014). A review of unsupervised feature learning and deep learning for time-series modeling. Pattern Recognition Letters.

[26] COCO Consortium. COCO - Common Objects in Context. (2016). Available at, http://mscoco.org/dataset/#detections-leaderboard. (Accessed: 14th October 2017).

[27] VanDussen Kelli L, Marinshaw Jeffrey M, Shaikh Nurmohammad, Miyoshi Hiroyuki, Moon Clara, Tarr Phillip I, Ciorba Matthew A, Stappenbeck Thaddeus S (2014). Development of an enhanced human gastrointestinal epithelial culture system to facilitate patient-based assays. Gut.

[28] Sato T, Clevers H (2013). Growing self-organizing mini-guts from a single intestinal stem cell: Mechanism and applications. Science.

